# Land use change has stronger effects on functional diversity than taxonomic diversity in tropical Andean hummingbirds

**DOI:** 10.1002/ece3.3813

**Published:** 2018-02-25

**Authors:** Boris A. Tinoco, Vinicio E. Santillán, Catherine H. Graham

**Affiliations:** ^1^ Department of Ecology and Evolution Stony Brook University Stony Brook NY USA; ^2^ Escuela de Biología Ecología y Gestión Universidad del Azuay Cuenca Ecuador; ^3^ Swiss Federal Research Institute WSL Birmensdorf Switzerland

**Keywords:** deforestation, disturbance, Ecuador, functional traits, montane forest, pollination services

## Abstract

Land use change modifies the environment at multiple spatial scales, and is a main driver of species declines and deterioration of ecosystem services. However, most of the research on the effects of land use change has focused on taxonomic diversity, while functional diversity, an important predictor of ecosystem services, is often neglected. We explored how local and landscape scale characteristics influence functional and taxonomic diversity of hummingbirds in the Andes Mountains in southern Ecuador. Data was collected in six landscapes along a land use gradient, from an almost intact landscape to one dominated by cattle pastures. We used point counts to sample hummingbirds from 2011 to 2012 to assessed how local factors (i.e., vegetation structure, flowering plants richness, nectar availability) and landscape factors (i.e., landscape heterogeneity, native vegetation cover) influenced taxonomic and functional diversity. Then, we analyzed environment – trait relationships (RLQ test) to explore how different hummingbird functional traits influenced species responses to these factors. Taxonomic and functional diversity of hummingbirds were positively associated with landscape heterogeneity but only functional diversity was positively related to native vegetation coverage. We found a weak response of taxonomic and functional diversity to land use change at the local scale. Environment‐trait associations showed that body mass of hummingbirds likely influenced species sensitivity to land use change. In conclusion, landscape heterogeneity created by land use change can positively influence hummingbird taxonomic and functional diversity; however, a reduction of native vegetation cover could decrease functional diversity. Given that functional diversity can mediate ecosystem services, the conservation of native vegetation cover could play a key role in the maintenance of hummingbird pollination services in the tropical Andes. Moreover, there are particular functional traits, such as body mass, that increase a species sensitivity to land use change.

## 1 INTRODUCTION

Land use change is one of the most important drivers of species loss and degradation of ecosystem services (Cardinale et al., 2012; Sala et al., 2000). The effects of land use change on diversity are usually measured based on taxonomic diversity, while functional traits, which are more likely to influence ecosystem services, are often not considered (Cadotte, Carscadden, & Mirotchnick, 2011; Mouillot, Graham, Villéger, Mason, & Bellwood, 2013). However, taxonomic and functional diversities are not always equally influenced by land use change (Luck, Carter, & Smallbone, 2013; McConkey & O'Farrill, 2015; Villéger, Miranda, Hernández, & Mouillot, 2010). For example, if assemblages contain species with similar functional roles, species loss will have a greater negative effect on taxonomic diversity than on functional diversity (Flynn et al., 2009). In contrast, if assemblages contain species with unique functional traits, the loss of a species can have greater consequences for functional diversity than for taxonomic diversity because the loss of a species could eliminate a functional role (Flynn et al., 2009). Here, we evaluate how taxonomic and functional diversities of hummingbirds are affected by ongoing land use change in the tropical Andes.

Land use change modifies environmental characteristics at multiple spatial scales (Tscharntke et al., 2012). At the landscape scale, anthropogenic activities modify the type and distribution of land cover in a landscape (Fahrig et al., 2011). A decrease in the coverage of the original vegetation in the landscape can negatively affect the persistence of habitat specialists (Betts, Forbes, & Diamond, 2007; Martensen, Ribeiro, Banks‐Leite, Prado, & Metzger, 2012). However, an increase in the number of land cover types in a landscape may sustain populations of species that use the resources provided by these novel types (Renjifo, 2001; Tscharntke et al., 2012). At the local‐habitat scale, a common result of land use change is the replacement of forest with more structurally simple vegetation dominated by smaller trees or pastures (Brawn, Robinson, & Thompson, 2001). In addition, there are often changes in the types and abundance of resources (Feinsinger et al., 1988; Hagen & Kraemer, 2010; Loiselle & Blake, 1994). Recent studies have demonstrated the value of measuring the types and diversity functional traits of species in a community to evaluate ecosystem functioning (Flynn et al., 2009; Mouillot et al., 2013). However, while extensive work has documented how landscape and local factors influence taxonomic diversity (e.g., Graham & Blake, 2001; Tinoco, Astudillo, Latta, Strubbe, & Graham, 2013), few studies have evaluated how these factors affect functional diversity, especially in species‐rich systems such as the tropical Andes (Tscharntke et al., 2008).

Hummingbirds are particularly suitable for the study of the effects of land use change on biodiversity. They are among the most species‐rich and abundant groups of birds in the tropical Andes (Rahbek & Graves, 2000). They also vary in morphology, habitat requirements, and foraging roles (Abrahamczyk & Kessler, 2014; Brown & Kodric‐Brown, 1979; Feinsinger & Colwell, 1978), resulting in high levels of local functional diversity (Graham, Parra, Tinoco, Stiles, & McGuire, 2012; Maglianesi, Blüthgen, Böhning‐Gaese, & Schleuning, 2015). As hummingbirds play a key role in the ecosystem as pollinators (Stiles, 1981), any effect of land use change on the functional diversity of this group can have consequences for the ecological functions they perform. While hummingbird taxonomic diversity is often considered relatively insensitive to land use change (Renjifo, 2001; Stouffer & Bierregaard, 1995), a recent study by Hadley, Frey, Robinson, and Betts (2017), in the lowlands of Costa Rica, found that species richness, relative abundance, and species with specialized morphologies can be sensitive to habitat loss. Nonetheless, whether hummingbird taxonomic and functional diversities respond equally to land use change remains an open question.

We collected data in six landscapes in the Andes of Ecuador along a land use gradient—from an almost intact landscape dominated by native vegetation to a landscape largely composed of cattle pastures to explore how local‐ and landscape‐scale characteristics influence both taxonomic and functional diversities of hummingbirds. Given the morphological variation across hummingbirds, we expected that functional diversity will be at least, if not more influenced by land use change than taxonomic diversity. Moreover, using extensive knowledge of functional traits in hummingbirds, we developed a set of predictions for how these traits may influence a hummingbird's response to land use change (Table 1) and tested those predictions by performing an analysis of environment–trait relationships.

**Table 1:** Description of functional traits and predictions of their influences on hummingbird species sensitivity to land use change

Functional trait	Functional influence	Prediction
Bill length	Hummingbirds with long bills have a narrow diet breath compared to hummingbirds with short bills (Maglianesi et al., 2014; Tinoco et al., 2017)	Hummingbirds with long bills will be negatively affected by land use change because they are less able to respond to changes in resource availability (Newbold et al., 2014)
Body mass	Heavier birds have smaller population sizes than lighter hummingbirds (Calder & Calder, 1995)	Heavier species will be sensitive to land use change because species with a small population size are often affected by land use change (Hadley et al., 2017; Henle et al., 2004)
Wing loading and width of wings	Low wing loading and narrow wings are related to trap‐lining behavior (sensu Feinsinger & Colwell, 1978) and increase the efficiency with which birds fly among patches of flowers (Stiles, 2008)	Species with low wing loading and narrow wings (i.e., trap‐liner species) will be more sensitive to land use change, which can result in unpredictable variation in the availability of their specialized nectar resources (Henle et al., 2004)
Tarsus length	Birds with longer tarsi tend to perch while foraging on flowers (Stiles, 2008)	Perching while feeding is influenced by flower architecture, because it requires floral structures with landing platforms (Miller, 1985). Land use change can alter the types of flowers available and could influence the use of perching as a foraging option in hummingbirds

## 2 METHODS

### 2.1 Study region

**Figure 1:**
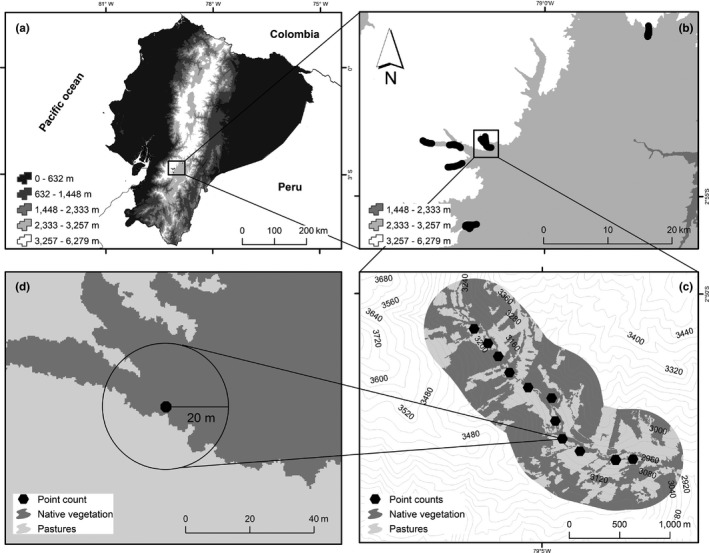
Map of the study area in the Andes of southern Ecuador where (a) is the country of Ecuador, (b) is our study region that contains our six landscape units, (c) is an example of a landscape unit with point counts, and (d) shows the local scale where vegetation plots were conducted

We conducted this study in the western Andes of the Azuay province in southern Ecuador between 3,000 and 3,300 m.a.s.l. (Figure 1). This region has a mean annual precipitation ranging from 1,100 to 1,800 mm, and monthly mean temperatures that range from 5 to 12°C (Celleri, Willems, Buytaert, & Feyen, 2007). Cattle pastures dominate the area with remnants of Andean native forest confined to steep slopes and along streams (White & Maldonado, 1991).

### 2.2 Study design

Our study design was hierarchical such that local‐scale plots are nested within landscapes (Figure 1). We chose six inter‐Andean linear valleys of approximately 300 ha in size within our study region (Figure 1). In each valley, we delineated an area of 2.2 km by 1 km (200 ha) referred to hereafter as a landscape unit (LU). The dimension of the LUs was chosen to fit the linear shape of the valleys, with the restriction that they were between 3,000 and 3,300 m.a.s.l. This elevation band covers most of the suitable habitat for forest birds in the study area because at higher elevations the vegetation transitions toward páramo grasslands, and at lower elevations, the landscape is mostly dominated by cattle pastures. The distances between LUs ranged from 2.5 to 34 km, and all valleys were partially isolated by steep slopes and páramo grasslands, which likely restricts most hummingbird movement to each valley. Limited movement was evidenced by the fact that there have only been three captures of hummingbirds between two adjacent valleys, Mazan and Llaviuco, during 8 years of systematic mist‐netting (Tinoco, unpublished data). Finally, the land use gradient in LUs varied from an almost intact valley, dominated by native vegetation, to a highly modified valley with a mosaic of pastures for cattle ranching, native vegetation remnants and exotic forests plantations (Table 2).

**Table 2:** Description of landscapes where hummingbirds were sampled in the Andes of southern Ecuador

Local name	Landscape composition			Landscape heterogeneity		Description of the type of anthropogenic alterations
	Native vegetation (%)	Pastures (%)	Exotic forest (%)	Edge density (m/Ha)	Landscape diversity	
Mazán	89.89	7.56	0.00	235.93	0.19	Native vegetation dominated
Llaviuco	77.02	22.98	0.00	466.91	0.35	Native vegetation with pastures
Culebrillas	76.62	18.45	4.93	521.25	0.38	Native vegetation dominated mosaic
Cubilán	60.86	39.14	0.00	772.95	0.48	Native vegetation dominated mosaic
Nero	54.63	38.08	7.30	361.27	0.55	Mixed used mosaic
Aurora	34.67	65.33	0.00	601.00	0.49	Pastures dominated

Within each LU, we positioned twelve 30‐m‐radius local‐scale plots (0.28 ha) at 200‐m intervals (Figure 1). We chose 200 m because it represents a distance greater than the daily movement of territorial hummingbirds (Dearborn, 1998; Paton & Carpenter, 1984) and allowed us to obtain replicate plots within LUs. However, we are aware of potential nonindependence of data among local‐scale plots located within LUs, which we accounted for in our modeling procedure (see Section 4).

This study was conducted in 2011 and 2012. We performed four surveys per LU each year between February and August. All valleys were surveyed during a period of about 7 days. During each survey, we gathered data on hummingbird and flower abundance.

### 2.3 Hummingbird sampling

We sampled hummingbirds using point counts in each of the 12 plots in each LU during each of our eight survey periods for a total of 288 counts. Each point count lasted 10 min, a time period that has been widely used to sample tropical bird communities (Blake & Loiselle, 2001; O'Dea, Watson, & Whittaker, 2004), because it maximizes the probability of registering most species, while decreasing the probability of double‐counting individuals (Esquivel & Peris, 2008; Smith et al., 1998). During each point count, two observers identified and counted all hummingbird individuals visually or acoustically detected in a fixed radius of 30 m. Hummingbirds flying over the plot were excluded. All the point counts within a LU were sampled on the same day between 06:00 hours and 10:00 hours, and on days without rain. We altered the starting time of each point count among survey periods to account for potential differences in bird detection among point counts due to time of day. We used the double‐observe method (software DOBSERV; Nichols et al., 2000) to estimate abundance. Using this method, detections from the two independent observers were combined to obtain a joint detection probability, which was used to correct the abundance estimates of each species in each point count for each survey period.

We obtained an accumulated annual species richness and a mean abundance per species in each plot per year (i.e., four survey periods). To explore the completeness of our estimates of annual species richness per plot, we compare our recorded values with the Chao 1 index, a nonparametric estimator of total richness (Chao, 1984). We recorded a high percentage of the total expected richness per plot according to the Chao 1 index (mean per plot 79.9% ± 2.03). Moreover, as the same method was applied across all plots, we can explore relative differences in hummingbird diversity across plots and LUs.

### 2.4 Landscape unit characteristics

We used aerial photographs 1:5,000 of the study area (SIGTIERRAS ‐ MAGAP, 2010) to quantify land cover types in each LU. The photographs were manually digitalized using ArcMap ver. 9.0 (ESRI, 2011) and classified into the following land cover types: native vegetation, cattle ranching pastures, and exotic forest. Native vegetation included both native forest and shrubs as the photographs did not permit finer classification. However, differences in vegetation structure within the native vegetation were minor compared to the differences with the other land cover types. In the study area, exotic forests are dominated by *Eucalyptus globulus*. Our classification was verified in the field by cross‐checking the actual vegetation with the mapped vegetation of each LU.

We calculated three landscape characteristics to describe land use change for each LU: percent native vegetation cover, and two measures of landscape heterogeneity: edge density and landscape diversity. Edge density, the number of edges in a landscape divided by the total landscape area, considers the spatial pattern of land cover types irrespective of their identity (Fahrig et al., 2011). Landscape diversity considers the number and proportion of each land cover type in the landscape and was estimated by the Simpson index (Fahrig et al., 2011). These three landscape characteristics were chosen because they are important predictors of biodiversity (Fahrig et al., 2011) and describe the gradient of land use in our study area (Table 2). LUs with low human influence had more native vegetation, low edge density, and low values of landscape diversity, while those with greater human influence had lower coverage of native vegetation, higher edge density, and higher values of landscape diversity.

### 2.5 Local plot habitat characteristics

From the center point of each plot located within LUs, we established four 20‐m‐long transects in each of the four cardinal directions. We quantified foliage height diversity, canopy cover, abundance of shrubs, and number of trees in different diameter at breast height (DBH) classes following a protocol commonly used in bird studies (James & Shugart, 1970). To measure foliage height diversity, we placed a 3‐m pole at 4‐m intervals along each transect and recorded the presence or absence of vegetation touching the pole at 0.5‐m intervals from 0 to 3 m. Above 3 m, we visually estimated the presence or absence of vegetation at 1‐m intervals to the top of the canopy which generally was less than 10 m tall. We calculated a Shannon diversity index to quantify foliage height diversity. We visually estimated the canopy cover using a scale of 1 to 5 (1 = 0%–19%, 2 = 20%–39%, 3 = 40%–59%, 4 = 60%–79%, 5 = 80%–100%). Foliage height diversity and canopy cover were averaged across all readings inside a vegetation plot (*n* = 20). The abundance of shrubs (plants with <3 cm of DBH) was obtained by counting the shrubs that contacted the extended arms of a person walking along each transect. Finally, all trees present inside the plot were counted and assigned to one of four different DBH categories: 3–8, 9–15, 16–23, 24–38, and >38 cm. In most cases (68.2%), trees of the largest DBH categories (24–38 and >38 cm) were the exotic species *E. globulus*.

To obtain a composite description of the vegetation structure in each plot, we used principal components analysis (PCA) with all the vegetation structure variables measured in each plot. PCI accounted for 54.2% of the variation and represented a gradient of plots dominated by open vegetation, to plots with closed vegetation (See Appendix Table S1). PCII depicted a gradient from plots with abundant large trees (mainly *Eucalyptus* trees) to plots dominated by shrubs and accounted for 20.9% of the variation (Appendix Table S1).

During each survey period in each plot, we sampled the richness of flowering plants and abundance of flowers. We included plant species that were observed to be used by hummingbirds during 405 hr of observation (Tinoco, Graham, Aguilar, & Schleuning, 2017). Plant species were identified by local experts from the Herbarium Azuay—University of Azuay in Cuenca, Ecuador. Flowering plant richness was the total number of plant species with open flowers. We measured the abundance of flowers by counting the number of open flowers of each plant. In cases where a complete count was not possible (e.g., trees, dense shrubs), we counted the number of flowers in a portion of the plant and estimated the total number of flowers by extrapolating the number of flowers in the sampled portion to the total area of the foliage covered with flowers.

Mean sugar production per flower of each species in our plots was obtained from recently opened flowers that were bagged for 24 hr to prevent access by hummingbirds, after which nectar was extracted with capillary tubes and sugar concentration measured with a handheld refractometer. Flowers were depleted of nectar before bagging (the number of flowers sampled and nectar production per species are provided in Appendix Table S2). Sugar production was calculated as the product of nectar volume in milliliters multiplied by sugar concentration (mg/ml) following the table provided by Kearns and Inouye (1993). Sugar production rates per plot and sampling period were obtained by multiplying the mean sugar production over 24 hr per flower with the total number of open flowers of the respective plant species. Flower abundance and sugar production rates were significantly correlated (*r* = .80, *p* < .01); therefore, we only included sugar production in further analysis because it is a direct measure of energy availability for pollinators (Potts, Vulliamy, Dafni, Ne'eman, & Willmer, 2003). Richness of flowering species and sugar production rates were averaged across survey periods within each year to obtain annual mean estimates of resource availability per plot.

### 2.6 Taxonomic and functional diversities

We calculated taxonomic and functional diversities of hummingbirds at each plot using the annual mean abundance of each species. We excluded species with less than three records across the study. Taxonomic diversity was calculated by the Simpson index. We used the following hummingbird functional traits to calculate functional diversity: body mass (weight of a live individual), bill length (length of the bill from base to tip), tarsus length (length from the outer bend of the tibiotarsal articulation to the base of the toes), wing loading (the ratio of body mass to wing area), and wing aspect ratio (the quotient of twice the square of the wing length divided by wing area). High wing loading represents a high body mass to wing area ratio, and a high aspect ratio denotes narrow wings. We only used morphological data for males because of their greater sample size. While some species in the studied community are sexually dimorphic, the standard deviation of a trait value within a species including measures of both sexes is much lower than the standard deviation of a trait across males of different species (Tinoco et al., 2017). Intraspecific variation related to sex is, therefore, unlikely to influence our results. Moreover, over 48% of our records in point counts were acoustic, from which it was impossible to sex individuals. However, we are aware that there could be behavioral differences between sexes in hummingbirds, and future research should explore if this variation influences functional diversity by studying behavior, movements, and landscape use at the individual level (Volpe, Robinson, Frey, Hadley, & Betts, 2016).

We calculated functional diversity using the Rao quadratic diversity index (Botta‐Dukát, 2005). The Rao index is equivalent to the Simpson diversity index when species completely differ in functional traits, a property that facilitates comparison between taxonomic and functional diversities. Given that we were interested in measuring functional diversity beyond that which is explained by body size, we used the residuals from linear regressions of total bill length and tarsus length against body mass as uncorrelated functional traits (Reist, 1985). The Rao index was obtained using the package FD (Laliberte & Legendre, 2010) in R (R Development Core Team, 2013).

### 2.7 Data analysis

To consider the hierarchical structure of our study design (plots nested within LUs), we used two approaches. First, we evaluate the importance of cross‐scale correlations between local and landscape scales in hummingbird diversity responses using variance partitioning analysis (Whittaker, 1984). Next, we explored the environmental correlates of diversity using linear mixed models, which incorporated the hierarchical structure of the design in the modeling (Zuur, Ieno, Walker, Saveliev, & Smith, 2009).

An important issue in studies that have a hierarchical spatial design, such as ours, is the potential cross‐scale correlations between scales (i.e., correlations between local and landscape scales). This issue could produce biological responses that are not independent between scales; therefore, we used variance partitioning analysis to test the effects of cross‐scale correlations (Whittaker, 1984). Variance partitioning analysis estimates the amount of the response variable that can be attributed independently to one scale, once the effect of the other scale is accounted for using partial multiple regressions (Cushman & McGarigal, 2002). The relative size of independent and shared variation measured by adjusted *R* ^2^ of partial multiple regressions is used to determine the relative importance of cross‐scale correlations (Cushman & McGarigal, 2002). Variance partitioning was implemented using the R package vegan (Oksanen et al., 2015).

The variance partitioning of species richness, taxonomic diversity, and functional diversity revealed that the independent contribution to variance of local and landscape scales was greater than their shared contribution (Appendix Table S3). This result indicates that is possible to explore the associations of landscape and local factors with hummingbird diversity without considering cross‐scale correlations, for which we used linear mixed models.

To examine the influence of landscape‐scale factors (landscape diversity, edge density, native vegetation cover) and local‐scale factors (PCI, PCII, flowering plant richness, sugar production) on species richness, taxonomic diversity, and functional diversity of hummingbirds, we used likelihood‐based linear mixed models. We included the identity of the LU as random factor to accommodate the hierarchical structure of our study design because local plots within a given landscape may be more similar to each other than plots in different LUs. We also included year as random factor to account for potential temporal correlation in surveys. All local‐ and landscape‐scale factors were used as fixed factors. We constructed models with all possible combinations of local‐ and landscape‐level factors, but did not include interactions because of our low degrees of freedom. Richness of flowering plants, sugar production, edge density, and landscape diversity were log‐transformed to improve normality. We evaluated the goodness of fit of the global model by estimating the marginal *R* ^2^ (the variance explained by the fixed factors alone) and conditional *R* ^2^ (the variance explained by both the fixed and random factors, following Nakagawa and Schielzeth (2013)). We used multimodel inference to compare and ranked the models by Akaike's information criterion corrected for small sample size (AICc) (Burnham & Anderson, 2002) using the R package MuMin (Barton, 2011). We obtained model average coefficients from the top selected models with ΔAICc <2. Statistical significance of a factor was inferred when its 95% confidence intervals (CI) excluded zero values (Burnham & Anderson, 2002). Further, as the pairwise spatial distances among LUs were not equal, we checked for spatial autocorrelation in the residuals of the global models by assessing the significance of Morans' I values using the package spdep in R (Bivand, 2013). None of the models revealed spatial autocorrelation.

Variation in functional diversity of a community can be influenced by changes in species composition (i.e., beta diversity) or differences in functional traits. We used RLQ analysis to explore associations between hummingbird functional traits and environmental characteristics, accounting for differences in species composition and abundance (Doledec, Chessel, terBraak, & Champely, 1996). RLQ analysis is a constrained ordination that maximizes the covariance between sites and species on the basis of environmental variables and species' traits. RLQ analysis uses three matrices: environment by sites (R); species abundances by sites (L); and trait values by species (Q). The analysis measures the strength of correlation between environmental variables (R) and traits (Q) mediated by species abundances (L). The significance of the costructure between the R and Q matrices was obtained by 999 Monte Carlo permutations where permutations on the rows of the R and Q matrices were compared to the observed total inertia. A probability of less than 0.05 of the observed inertia was considered significant costructure between R and Q matrices. See details of this analysis in Doledec et al. (1996).

We performed RLQ analysis separately for the landscape and local scales. At the landscape scale, we used native vegetation cover, landscape diversity, and edge density in R, annual mean abundance per plot in L, and our five hummingbird functional traits in Q matrix. At the local scale, we used PCI, PCII, sugar production and richness of flowering plants per plot in R, and the same L and Q matrices as used in the landscape analysis. RLQ was implemented in the package ade4 (Dray & Dufour, 2007) in R (R Development Core team 2013).

## 3 RESULTS

We recorded 15 hummingbird species during our 576 point counts (Appendix Table S4). The most abundant species were *Metallura tyrianthina*,* Eriocnemis luciani*, and *Coeligena iris*. Mean hummingbird species richness per point count among LUs across years varied from 3.66 in the less disturbed LU (Mazan) to 2.54 in the most disturbed LU (Aurora) (Table 3). Species turnover among LUs, measured by Bray–Curtis distance, varied from 0.17 to 0.50 (Table 3); the two least altered LUs, Mazan and Llaviuco, had the most similar species composition, while Cubilán, an intermediately altered LU, and Aurora, the most altered LU, were most different from the other LUs (Table 3).

**Table 3:** Mean bird species richness per point count and Bray–Curtis pairwise dissimilarity distance across six landscapes with different levels of land use change in the south central Andes of Ecuador

Mean species richness	Low level of land use change		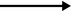		High level of land use change	
	Mazan	Llaviuco	Culebrillas	Cubilán	Nero	Aurora
Mazán (3.66 ± 1.65)	—	0.17	0.26	0.49	0.29	0.27
Llaviuco (3.13 ± 1.14)			0.3	0.46	0.23	0.35
Culebrillas (3.20 ± 1.38)				0.46	0.28	0.24
Cubilán (2.91 ± 0.92)					0.38	0.50
Aurora (2.54 ± 0.97)						0.38
Nero (3.45 ± 1.25)						—

Mean values and species composition for dissimilarity calculations were obtained by averaging data from 12 point counts and eight sampling periods per valley.

There was a large variation in the functional traits of hummingbird species (Appendix Table S5). *Chaetocercus mulsant* and *Ramphomicron microrhynchum* had body mass and bill lengths several orders smaller than *Pterophanes cyanopterus* and *Ensifera ensifera*. *Chaetocercus mulsant* and *R. microrhynchum* also had high wing loading, while species with low wing loading included *M. tyrianthina*,* Aglaeactis cupripennis*, and *P. cyanopterus*. Species with narrow wings, which have a higher aspect ratio, were *E. luciani* and *M. baroni*, while *M. tyrianthina* and *Lesbia nuna* had broad wings (Appendix Table S5).

### 3.1 Hummingbird diversity

The global linear mixed model of hummingbirds' species richness explained 18% of the variation in the data (marginal *R* ^2^ = .15, conditional *R* ^2^ = .18). Hummingbirds' species richness was significantly positively associated with the local‐scale factor flowering plant richness (Table 4). Around 26% of the variation in taxonomic diversity was explained by the global linear mixed model (marginal *R* ^2^ = .18, conditional *R* ^2^ = .26), and it varied positively with richness of flowering plants and the landscape factor, edge density (Table 4). The global linear mixed model of functional diversity explained 23% of the variation in the data (marginal *R* ^2^ = .21, conditional *R* ^2^ = .23). At the local scale, functional diversity increased with richness of flowering plants and was negatively associated with open plots (PCI) (Table 4); at the landscape scale, edge density, landscape diversity, and coverage of native vegetation were all positively associated with functional diversity (Table 4).

**Table 4:** Effects of different local (PCI, PCII, richness of flowering plants, sugar production) and landscape (native vegetation coverage, landscape diversity, edge density) factors on (A) species richness, (B) taxonomic diversity, and (C) functional diversity of hummingbirds across six landscapes (LUs) in the south central Andes of Ecuador

Factor	β	*SE*	95% CI		
			Lower	Upper	RIV
(A) Species richness					
PCI	−0.01	0.03	−0.16	0.05	0.15
PCII	0.05	0.06	−0.02	0.20	0.57
**Richness of flowering plants**	0.10	0.05	0.01	0.20	0.97
Sugar production	0.03	0.05	−0.02	0.17	0.44
Landscape diversity	−0.10	0.13	−0.40	0.10	0.65
Edge density	0.01	0.04	−0.06	0.21	0.14
Native vegetation cover	−0.04	0.12	−0.46	0.25	0.34
(B) Taxonomic diversity					
PCI	−0.01	0.02	−0.04	0.00	0.64
**Richness of flowering plants**	0.04	0.01	0.01	0.05	1
Sugar production	0.00	0.01	−0.01	0.04	0.34
Landscape diversity	−0.06	0.04	−0.01	0.01	1
**Edge density**	0.04	0.01	0.01	0.06	1
Native vegetation cover	−0.03	0.04	−0.13	0.02	0.5
(C) Functional diversity					
**PCI**	−0.16	0.04	−0.26	−0.07	1
PCII	−0.01	0.03	−0.13	0.05	0.17
**Richness of flowering plants**	0.06	0.04	0.00	0.14	0.83
Sugar production	0.02	0.03	−0.02	0.12	0.43
**Landscape diversity**	0.30	0.14	0.02	0.58	1
**Edge density**	0.14	0.05	0.03	0.25	1
**Native vegetation cover**	0.48	0.15	0.18	0.78	1

Given are standardized averaged estimates (β), their unconditional standard errors (*SE*), 95% confidence intervals (95% CI), and the relative variable importance (RVI) in the set of best models (ΔAICc values <2). Factors with significant effects (estimates for which 95% confidence intervals do not overlap zero) are highlighted in bold.

### 3.2 Trait–environment relationships

**Figure 2:**
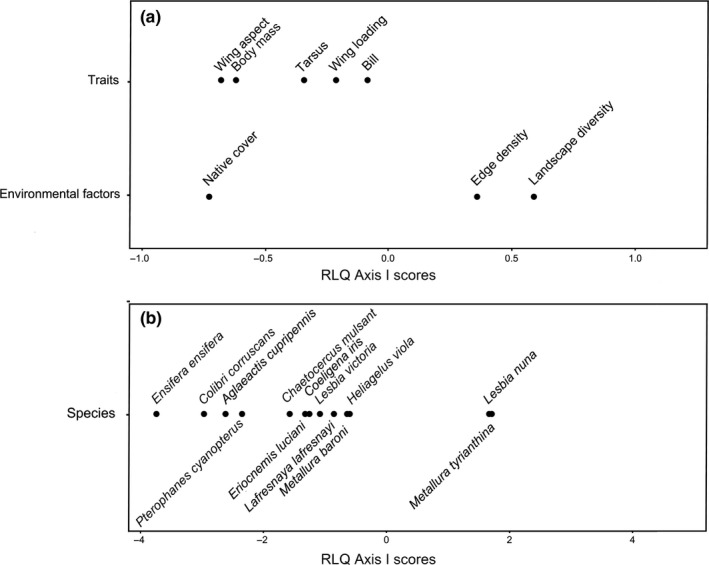
Graphical depiction of the first axis of an RLQ analysis for (a) functional traits and (b) environmental variables at the landscape scale. Position of scores relative to the origin indicates their contribution to RLQ axis, and relative position of scores along the axis indicates associations between functional traits and environmental variables. Species are plotted within functional trait space

RLQ analysis identified a significant relationship between landscape variables and species traits (Permutation test; *p* = .002). We only considered Axis I because it accounted for 95.3% of the variance in the RLQ analysis. Important variables included coverage of native vegetation with a positive loading, and landscape diversity and edge density which had negative loadings, depicting a gradient from high to low levels of human land use change (Figure 2). The positive loadings of body mass, wing aspect ratio, and relative tarsus length indicated an association of these traits with less altered LUs (Figure 2). When species were plotted in functional traits space, we found that species positively associated with characteristics of less altered LUs were *E. ensifera*,* P. cyanopterus*,* C. iris*, and *A. cupripennis* (Figure 2), while species that likely preferred characteristics of more altered LUs included *M. tyrianthina* and *L. nuna*.

**Figure 3:**
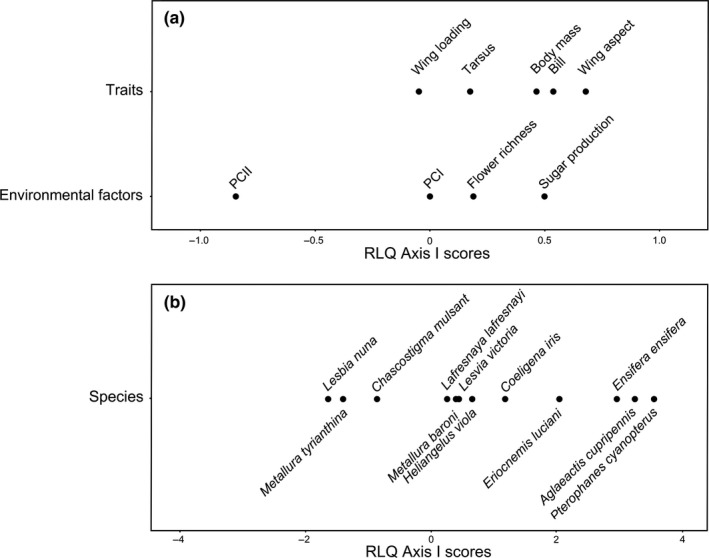
Graphical depiction of the first axis of an RLQ analysis for (a) functional traits and (b) environmental variables at the local scale. Position of scores relative to the origin indicates their contribution to RLQ axis, and relative position of scores along the axis indicates associations between functional traits and environmental variables. Species are plotted within functional trait space

In the local‐scale RLQ analysis, there was a significant association between local factors and species traits (Permutation test; *p* = .001) with 82.4% and 10.1% of the variation was accounted for by Axis I and Axis II, respectively. We only interpreted Axis I, which was mainly described by PCII with a negative loading, and sugar production with a positive loading (Figure 3), thus representing a gradient from plots with large trees (mainly *Eucalyptus*), and less sugar availability on the left side of the ordination to plots characterized by the presences of shrubs and greater sugar availability in the right side. Body mass, wings aspect ratio, and bill length were associated with plots dominated by shrubs and high sugar availability (Figure 3). Plotting species within functional traits space revealed that species associated with plots with abundant shrubs and high resource availability included *P. cyanopterus*,* A. cupripennis*, and *C. iris*, while species associated with plots with the presence of large trees and low sugar availability included *L. nuna* and *M. tyrianthina* (Figure 3).

## 4 DISCUSSION

Land use change modifies the environment at multiple spatial scales, which could differentially affect facets of biodiversity (Frishkoff et al., 2014; Luck et al., 2013). Here, we used a hierarchical study design to evaluate the effects of land use change at the landscape and local scales of both taxonomic and functional diversities of hummingbirds in the tropical Andes. At the landscape scale, both facets of diversity of hummingbirds were positively associated with an increase in landscape heterogeneity. However, unlike taxonomic diversity, functional diversity was positively associated with native vegetation cover, which indicates that trait diversity may be more affected by land use change than taxonomic diversity (Flynn et al., 2009). At the local scale, we found a weak response of taxonomic and functional diversities to land use change; these results suggest that landscape‐scale factors could be more important than local‐scale factors in mediating how hummingbirds respond to anthropogenic disturbance. Moreover, we found that large body mass, narrow wings, and long tarsi increased hummingbirds' sensitivity to land use change. Taken together, these results indicate that different facets of diversity and environmental factors at multiple scales should be considered when evaluating how anthropogenic activities influence biodiversity (Cadotte et al., 2011; Frishkoff et al., 2014; Monnet et al., 2014).

### 4.1 Hummingbird diversity

Our results support the common finding that landscape heterogeneity, quantified here as edge density and landscape diversity, promotes increased animal diversity in human‐modified landscapes (Fahrig et al., 2011; Tscharntke et al., 2012). Edge density was positively associated with both taxonomic and functional diversities. Edge density, related to the amount of habitat boundaries in the landscape, may promote species' movements between complementary habitats (Hughes, Daily, & Ehrlich, 2002), and often, edges contain plant species that provide abundant nectar resources (Hagen & Kraemer, 2010; Tscharntke et al., 2008). Landscape diversity, positively associated with functional diversity, can increase niche space (Fahrig et al., 2011), thus promoting the accumulation of species with different traits and habitat requirements (Haslem & Bennett, 2008; Perović et al., 2015).

In addition, functional diversity increased with native vegetation cover, indicating that functional diversity could be more sensitive to land use change than taxonomic diversity. Taxonomic and functional diversities may have different responses to land use gradients because altered environments can set limits to the ranges of traits of coexisting species, and thus constrain the amount of functional variation possible in assemblages (Cadotte et al., 2011). For example, species richness often remains the same after anthropogenic disturbance because, while some forest species may be lost, generalist species often colonize these disturbed landscapes (Graham & Blake, 2001; Renjifo, 2001). In our case, species with distinct functional traits, including *E. ensifera* and *P. cyanopterus*, were uncommon in more disturbed landscapes while species with generalized functional traits such as *L. nuna* and *M. tyrianthina* were more common. This change in abundance patterns likely explains why functional diversity, but not species richness or taxonomic diversity, declined as the amount of native vegetation coverage in the landscape declined.

Our studied landscapes presented continuous variation in the amount of native vegetation cover and landscape heterogeneity; thus, our results are the product of the combined effects of these two factors. A more complete understanding of the mechanism influencing biodiversity responses to land use change that distinguishes the independent effects of loss of native vegetation and landscape heterogeneity would require replicates of landscapes with the same levels of habitat loss and heterogeneity (see review by Hadley & Betts, 2016). This study design was not possible in our region because there is a long history of anthropogenic disturbance which limits by the number of LUs at the same elevation that contain montane forest (White & Maldonado, 1991). In addition, there can be intraspecific behavioral differences between male and female hummingbirds in their responses to land use change (Feinsinger & Colwell, 1978), which could be obscured with species‐level analyses. Studies, potentially using marked individuals so sex can be determined, should aim to disentangle the influence of land use change on the complete functional diversity of hummingbirds in the tropical Andes.

At the local scale, variation across all measures of hummingbird diversity was associated with richness of flowering plants. Trait matching between bill length and corolla length influences hummingbird resource use (Weinstein & Graham, 2017; Wolf, 1978); therefore, if an increase in plant species richness corresponds to an increase in plants with morphologically different corollas, potentially more hummingbird species can coexist through resource partitioning (Abrahamczyk & Kessler, 2014; Feinsinger & Colwell, 1978). A positive correlation between plant and pollinator species richness has also been reported in plant–insect pollination systems (Fründ, Linsenmair, & Blüthgen, 2010; Weiner, Werner, Linsenmair, & Blüthgen, 2011). Future work should explore the relationship between functional diversity of flowers and functional diversity of pollinators.

Variables of the vegetation structure at the local scale did not appear to influence hummingbird taxonomic richness or diversity; however, functional diversity was higher in plots with more open vegetation. This result is consistent with the apparent importance of the landscape factors edge density and landscape diversity. Open areas are often of high quality because of the presence of plant species with flowers that produce abundant nectar (Costa & Magnusson, 2003). In our study area, plant species such as *Oreocalllis grandiflora* and *Barnadesia arborea* are pioneer colonizers with flowers that attract many species of hummingbirds (Tinoco et al., 2017). In addition, the strong association found between all three landscape factors and hummingbird functional diversity suggests that relationship between local‐scale factors and hummingbirds might be largely dependent on the landscape context (Renjifo, 2001; Tscharntke et al., 2012). Some hummingbird species have daily movements of more than 1 km (Hadley & Betts, 2009) and may exploit resources across different land cover types; for example, a recent study by Volpe et al. (2016) found that the presence of the hummingbird *Phaethornis guy* in small forest patches is highly dependent in the connectivity of the native forest at the landscape scale. Thus, the use of different land cover types in a landscape may undermine negative effects of land use change at small local scales for many hummingbird species.

### 4.2 Trait–environment relationships

As predicted (Table 1), body mass, tarsus length, and narrow wing aspect appeared to be negatively influenced by land use change at the landscape scale. In our study, *P. cyanopterus*,* E. ensifera*, and *E. luciani* are among those species with high body mass, narrow wings, and long tarsi that negatively responded to land use change. Hadley et al. (2017) found that hummingbirds having specialized morphologies (i.e., large body size) can be sensitive to habitat loss and fragmentation. Increased sensitivity to land use change in heavier species has been found elsewhere (Gage, de Brooke, Symonds, & Wege, 2004; Newbold et al., 2014) and has been attributed to the correlation between body mass and demographic parameters, such as small population size and low reproduction rates (Henle, Davies, Kleyer, Margules, & Settele, 2004). Species with these demographic characteristics are often prone to extinction via environmental and demographic stochasticity (Lande, 1993). Moreover, heavier hummingbirds also had longer bills (note that our measure of bill length accounted for body mass which may be why this trait is not strongly associated with the RLQ plot), which could increase hummingbirds′ sensitivity to land use change because longer billed species have more specialized diets (Tinoco et al., 2017) and may decline because their resources are often less common in disturbed environments (Newbold et al., 2014).

Narrow‐winged species were also associated with less disturbed landscapes. This trait confers reduced power requirements during flight, a characteristic that would benefit trap‐lining behavior in hummingbirds (Feinsinger & Colwell, 1978). Species that forage for resources that are patchy in the environment are thought to be more sensitive to land use change because of unpredictable variation in their specialized resources (Gibb et al., 2006; Henle et al., 2004). Trap‐lining hummingbirds can also be affected by land use change if they avoid crossing open areas in their foraging routes (Hadley et al., 2017). Nonetheless, we acknowledge that flying behavior in hummingbirds is defined by a complex group of parameters influencing flying aerodynamics (Altshuler, Stiles, & Dudley, 2004), and more research should be carried out to directly measure the influence of wing shape and other traits in the foraging behavior of hummingbirds. Finally, the positive association between tarsus length and more pristine LUs could be related to the types of flowers available across the land use gradient. In hummingbirds, species with long tarsi frequently perch on flowers for feeding (Stiles, 2008), a behavior dependent on the availability of flowers with landing structures (Miller, 1985).

At the local scale, the RLQ indicated that hummingbirds with narrow wings, large body mass, and large bills were more common in plots with high resource abundance. Species that have those functional traits included *P. cyanopterus*,* A. cupripennis*, and *E. ensifera*. Moreover, we found that plots with large trees and low abundance of sugar were associated with small hummingbirds with short bills. These plots likely corresponded to areas with an important presence of the exotic *Eucalyptus* trees, where species like *L. nuna* and *M. tyrianthina* can be abundant. Other studies have reported that *Eucalyptus* trees can sustain hummingbird populations (Montaldo, 1984; Renjifo, 2001), but as found here, this observation might apply only to species with particular traits, such as small body size and short bill. More generally, the result that the association between some hummingbird traits and the environment were as predicted highlights the importance of trait‐based approaches for understanding the factors that influence species responses to land use change.

### 4.3 Conservation implications

Heterogeneous landscapes with significant coverage of native vegetation correspond to intermediately disturbed landscapes. While hummingbird functional diversity might benefit from some level of land use change, as found here, there is likely a threshold beyond which heterogeneity can negatively affect diversity. This is because an increase in heterogeneity leads to a loss of native vegetation (Betts et al., 2007; Cerezo, Conde, & Poggio, 2011), producing a negative impact to species with unique functional roles (Carrara et al., 2015).

Functional diversity of pollinators can increase fruit productivity in plants (Hoehn, Tscharntke, Tylianakis, & Steffan‐Dewenter, 2008); thus, maintenance of this diversity might be key for sustaining high‐quality pollination services (Rader, Bartomeus, Tylianakis, & Laliberté, 2014). In the future, it will be important to directly test how changes in the functional diversity of hummingbirds can alter pollination services. Nonetheless, this study points out the importance of the maintenance of native vegetation to promote high levels of functional diversity of hummingbirds in heterogeneous agricultural landscapes. Cattle ranching is one of the main causes of the reduction in native vegetation coverage across the tropical Andes (Viña & Cavelier, 1999), but several management actions could be implemented to both retain the commercial value of cattle ranching activities and promote hummingbird functional diversity. The protection of native vegetation remnants, the incorporation of native vegetation hedgerows, the maintenance of native vegetation along rivers, and the use of native flowering plants on fences are all important practices that could contribute to the conservation of hummingbird taxonomic and functional biodiversity and the pollination services they provide.

## CONFLICT OF INTEREST

None declared.

## AUTHOR CONTRIBUTIONS

B.A.T. and C.H.G. designed the study. B.A.T. collected field data. B.A.T. and V.E.S. performed data analysis. B.A.T. and C.H.G. wrote the manuscript. All authors critically read and commented on the manuscript.
