# Supplementary material for: Land use change has stronger effects on functional diversity than taxonomic diversity in tropical Andean hummingbirds
Source: Ecol Evol. 2018 Feb 25;8(6):3478–90. doi: 10.1002/ece3.3813 (PMC5869371; doi:10.1002/ece3.3813)
Supplement: Supplementary file 1 [file ECE3-8-3478-s001.docx]

**Supporting Information**

**Table S1.** Principal components analysis of the vegetation structure of plots sampled across six landscapes in the southern Andes of Ecuador.

**Table S2**. Nectar characteristics of all plant species used by the hummingbird species recorder across six landscapes in the southern Andes of Ecuador.

**Table S3.** Results of variance partitioning analysis of local and landscapes scales in the variance of species richness, taxonomic diversity and functional diversity of hummingbirds in the southern Andes of Ecuador.

**Table S4.** List of hummingbird species recorded during the study period in the southern Andes of Ecuador.

**Table S5**. Measures of functional traits of male hummingbirds recorded in this study in the southern Andes of Ecuador.

**APPENDIX**

**Table S1.** Principal components analysis of the vegetation structure of plots sampled across six landscapes in the southern Andes of Ecuador. The highest factor loadings are in bold type.

| Variables | PC1 | PC2 |
| --- | --- | --- |
| Canopy cover | **1.39** | -0.30 |
| Foliage height diversity | **1.36** | -0.46 |
| Average height of canopy | 0.98 | **1.13** |
| Abundance of shrubs | **1.23** | **-0.92** |
| Abundance of trees 3-8 cm DBH | **1.40** | -0.59 |
| Abundance of trees 9-15 cm DBH | **1.44** | -0.22 |
| Abundance of trees 16-23 cm DBH | **1.33** | 0.18 |
| Abundance of trees 24-38 cm DBH | **1.04** | **1.02** |
| Abundance of trees ˃ 39 cm DBH | 0.76 | **1.18** |

**Table S2**. Nectar characteristics of all plant species used by the hummingbird species recorder across six landscapes in the southern Andes of Ecuador.

| Species | Nectar Volume | |  | Sugar Concentration | |  | Nectar production |
| --- | --- | --- | --- | --- | --- | --- | --- |
|  | (*u*l 24h¯1) | |  | (%) | |  | sugar mg¯24h per flower |
|  | N | Mean ( ±SE) |  | N | Mean ( ±SE) |  | Mean |
| *Alloplectus peruvianus* | 11 | 0.04 (±0.02) |  | 9 | 14.5 (±2.4) |  | 0.01 |
| *Barnadesia arborea* | 15 | 9.22 (±1.5) |  | 21 | 19.15 (±0.6) |  | 1.91 |
| *Berberis lutea* | 15 | 0.62 (±0.04) |  | 16 | 25.69 (±1.7) |  | 0.18 |
| *Bomarea sp* | 7 | 2.33 (±0.89) |  | 7 | 2.33 (±0.4) |  | 0.05 |
| *Brachyotum confertum* | 31 | 14.48 (±3.13) |  | 30 | 14.62 (±0.7) |  | 2.24 |
| *Bromelia sp2* | 9 | 1.5 (±0.22) |  | 16 | 10.63 (±0.5) |  | 0.17 |
| *Bromelia sp3* | 21 | 16.42 (±2.58) |  | 21 | 14.52 (±1.2) |  | 2.52 |
| *Brugmansia sanguinea* | 10 | 29.23 (±10.17) |  | 9 | 20.89 (±1.8) |  | 6.63 |
| *Cavendishia bracteata* | 18 | 0.14 (±0.04) |  | 7 | 16.31 (±1.3) |  | 0.02 |
| *Centropogon sp.* | 10 | 6.14 (±1.89) |  | 10 | 12.2 (±0.7) |  | 0.78 |
| *Fuchsia cf. vulcanica* | 44 | 6.39 (±1.74) |  | 22 | 19.8 (±1.4) |  | 1.37 |
| *Gaiadendron punctatum* | 5 | 0.09 (±0.01) |  | 3 | 31 (±1.4) |  | 0.03 |
| *Gaultheria erecta* | 15 | 0.23 (±0.05) |  | 8 | 13.9 (±1.8) |  | 0.03 |
| *Macleania rupestris* | 46 | 10.81 (±1.59) |  | 39 | 17.5 (±0.8) |  | 2.02 |
| *Mutisia lemanni* | 13 | 34.7 (±9.43) |  | 21 | 19.5 (±0.9) |  | 7.30 |
| *Oreocallis grandiflora* | 48 | 18.69 (±1.65) |  | 42 | 14.8 (±0.4) |  | 2.93 |
| *Palicuorea sp* | 10 | 0.94 (±1.33) |  | 10 | 14.4 (±0.6) |  | 0.14 |
| *Passiflora cumbalensis* | 5 | 142.51 (±29.45) |  | 9 | 23 (±1.4) |  | 35.88 |
| *Rubus floribundus* | 5 | 2.44 (±0.56) |  | 1 | 50 (±NA) |  | 1.50 |
| *Salvia corrugate* | 46 | 2 (±0.33) |  | 22 | 11.9 (±1.8) |  | 0.25 |
| *Salvia hirta* | 17 | 6.36 (±1.78) |  | 18 | 18.1 (±1.2) |  | 1.23 |
| *Saracha quitensis* | 14 | 13.29 (±2.54) |  | 19 | 12.5 (±0.9) |  | 1.74 |
| *Tillandsia complanata* | 9 | 1.49 (±0.22) |  | 9 | 10.6 (±0.7) |  | 0.17 |
| *Tristerix longebracteatus* | 30 | 4.56 (±0.8) |  | 18 | 18.9 (±0.6) |  | 0.93 |
| *Verbesina latisquama* | 15 | 0.69 (±0.05) |  | 18 | 22.1 (±0.5) |  | 0.17 |
| *Viola arguta* | 16 | 4.08 (±0.73) |  | 14 | 12.5 (±1.1) |  | 0.54 |

Table S3. Results of variance partitioning analysis that explored the unique and shared variation explained by local and landscape scales based on A) Species richness, B) Taxonomic diversity, and C) Functional diversity of hummingbirds across six landscapes in the southern Andes of Ecuador.

|  | **Adjusted R^2^** |
| --- | --- |
| **A) Species Richness** |  |
| Local Scale | 0.08 |
| Landscape Scale | 0.05 |
| Local Scale + Landscape Scale | 0.04 |
| Residuals | 0.83 |
|  |  |
| **B) Taxonomic diversity** |  |
| Local Scale | 0.07 |
| Landscape Scale | 0.06 |
| Local Scale + Landscape Scale | <0.01 |
| Residuals | 0.87 |
|  |  |
| **C) Functional diversity** |  |
| Local Scale | 0.13 |
| Landscape Scale | 0.1 |
| Local Scale + Landscape Scale | <0.01 |
| Residuals | 0.77 |

**Table S4.** List of hummingbird species recorded during the study period in the southern Andes of Ecuador. Values represent annual averages per point count across all the landscapes.

| Species name | Year | | |
| --- | --- | --- | --- |
|  | 2011 |  | 2012 |
| *Aglaeactis cupripennis* | 0.25 |  | 0.40 |
| *Chaetocercus mulsant* | 0.03 |  | 0.02 |
| *Coeligena iris* | 0.62 |  | 0.49 |
| *Colibri coruscans* | 0.25 |  | 0.05 |
| *Ensifera ensifera* | 0.02 |  | 0.06 |
| *Eriocnemis luciani* | 0.51 |  | 0.74 |
| *Eriocnemis vestita* | 0.00 |  | 0.05 |
| *Heliangelus viola* | 0.25 |  | 0.28 |
| *Lafresnaya lafresnayi* | 0.36 |  | 0.41 |
| *Lesbia nuna* | 0.42 |  | 0.28 |
| *Lesbia victoriae* | 0.12 |  | 0.03 |
| *Metallura baroni* | 0.06 |  | 0.02 |
| *Metallura tyrianthina* | 1.92 |  | 2.03 |
| *Pterophanes cyanopterus* | 0.06 |  | 0.09 |
| *Ramphomicron microrhynchum* | 0.00 |  | 0.03 |

**Table S5**. Measures of functional traits of male hummingbirds recorded in this study.

| Species | Body mass | |  | Total culmen | |  |  | Wing aspect ratio | |  | Wing loading | |  | Tarsus | |
| --- | --- | --- | --- | --- | --- | --- | --- | --- | --- | --- | --- | --- | --- | --- | --- |
|  | N | Mean (SE) gr |  | N | Mean (SE) mm |  |  | N | Mean (SE) |  | N | Mean (SE) g/cm^3^ |  | N | Mean (SE) mm |
| *Aglaeactis cupripennis* | 21 | 8.21 (±0.14) |  | 25 | 21.5 (±0.32) |  |  | 17 | 7.79 (±0.10) |  | 17 | 0.181(±0.01) |  | 17 | 7.78 (±0.20) |
| *Chaetocercus mulsant** | 24 | 3.58 (±0.03) |  | 24 | 19.5 (±0.15) |  |  | 24 | 7.72 (±0.10) |  | 24 | 0.368 (±0.01) |  | 7 | 4.33 (±0.02) |
| *Colibri coruscans* | 25 | 8.21 (±0.07) |  | 34 | 28 (± 0.54) |  |  | 25 | 7.82 (±0.05) |  | 25 | 0.21 (±0.01) |  | 25 | 6.22 (±0.28) |
| *Coeligena iris* | 5 | 7.08 (±0.11) |  | 7 | 31.81 (± 1.00) |  |  | 3 | 7.39 (±0.51) |  | 3 | 0.212 (±0.01) |  | 8 | 7.44 (±0.15) |
| *Ensifera ensifera** | 4 | 9.68 (±0.27) |  | 4 | 83.92 (± 1.33) |  |  | 4 | 7.94 (±0.16) |  | 4 | 0.254 (±0.01) |  | 4 | 6.8 (±0.11) |
| *Eriocnemis luciani* | 10 | 6.12 (±0.13) |  | 14 | 24.98 (± 0.40) |  |  | 1 | 7.9 (±NA) |  | 1 | 0.233 (±NA) |  | 13 | 7.79 (±0.20) |
| *Eriocnemis vestita** | 25 | 4.86 (±0.04) |  | 25 | 21.38 (± 0.17) |  |  | 25 | 7.14 (±0.05) |  | 25 | 0.2 (±0.01) |  | 25 | 5.7 (±0.05) |
| *Heliangelus viola* | 12 | 5.63 (±0.11) |  | 8 | 18.08 (± 0.78) |  |  | 6 | 7.39 (±0.32) |  | 6 | 0.246 (±0.01) |  | 8 | 7.29 (±0.19) |
| *Lafresnaya lafresnayi* | 19 | 5.69 (±0.07) |  | 18 | 29.69 (±0.26) |  |  | 19 | 7.63 (±0.05) |  | 19 | 0.216 (±0.01) |  | 18 | 5.62 (±0.01) |
| *Lesbia nuna** | 7 | 3.66 (±0.05) |  | 7 | 12.66 (± 0.21) |  |  | 7 | 6.82 (±0.06) |  | 7 | 0.198 (±0.01) |  | 7 | 5.64 (±0.08) |
| *Lesbia victoriae** | 11 | 5.29 (±0.05) |  | 11 | 17.58 (±0.17) |  |  | 11 | 7.44 (±0.07) |  | 11 | 0.226 (±0.01) |  | 11 | 6.25 (±0.07) |
| *Metallura baroni* | 4 | 4.57 (±0.08) |  | 3 | 17.77 (±0.62) |  |  | 2 | 7.86 (±0.52) |  | 2 | 0.248 (±0.01) |  | 3 | 7.07 (±0.55) |
| *Metallura tyrianthina* | 35 | 3.79 (±0.04) |  | 42 | 14.83 (±0.15) |  |  | 35 | 7.09 (±0.06) |  | 35 | 0.161 (±0.01) |  | 40 | 6.72 (±0.09) |
| *Pterophanes cyanopterus* | 6 | 10.37 (±0.31) |  | 6 | 36.45 (± 1.01) |  |  | 6 | 7.55 (±0.09) |  | 6 | 0.147 (±0.01) |  | 6 | 7.84 (±0.14) |
| *Ramphomicron microrhynchum** | 15 | 3.67 (±0.06) |  | 15 | 10.21 (±0.25) |  |  | 15 | 7.5 (±0.10) |  | 15 | 0.234 (±0.01) |  | 15 | 6.07 (±0.04) |

* symbol denotes species for which morphology data was obtained from Gary Stiles personal data base. Stiles contact information: Instituto de Ciencias Naturales, Universidad Nacional de Colombia, Bogotá, Colombia.
